# Comparing the mapping between EQ-5D-5L, EQ-5D-3L and the EORTC-QLQ-C30 in non-small cell lung cancer patients

**DOI:** 10.1186/s12955-016-0455-1

**Published:** 2016-04-12

**Authors:** Iftekhar Khan, Steve Morris, Nora Pashayan, Bashir Matata, Zahid Bashir, Joe Maguirre

**Affiliations:** Clinical Trials Unit & Department of Health Economics, University of Surrey, Guilford, UK; Department of Applied Health Research, University College London, London, UK; Liverpool Heart and Chest Hospital, Liverpool, UK; University of Birmingham, Cancer School, Birmingham, UK

## Abstract

**Background:**

Several mapping algorithms have been published with the EORTC-QLQ-C30 for estimating EQ-5D-3L utilities. However, none are available with EQ-5D-5L. Moreover, a comparison between mapping algorithms in the same set of patients has not been performed for these two instruments simultaneously. In this prospective data set of 100 non-small cell lung cancer (NSCLC) patients, we investigate three mapping algorithms using the EQ-5D-3L and EQ-5D-5L and compare their performance.

**Methods:**

A prospective non-interventional cohort of 100 NSCLC patients were followed up for 12 months. EQ-5D-3L, EQ-5D-5L and EORTC-QLQ-C30 were assessed monthly. EQ-5D-5L was completed at least 1 week after EQ-5D-3L. A random effects linear regression model, a beta-binomial (BB) and a Limited Variable Dependent Mixture (LVDM) model were used to determine a mapping algorithm between EQ-5D-3L, EQ-5D-5L and QLQ-C30. Simulation and cross validation and other statistical measures were used to compare the performances of the algorithms.

**Results:**

Mapping from the EQ-5D-5L was better: lower AIC, RMSE, MAE and higher R^2^ were reported with the EQ-5D-5L than with EQ-5D-3L regardless of the functional form of the algorithm. The BB model proved to be more useful for both instruments: for the EQ-5D-5L, AIC was –485, R^2^ of 75 %, MAE of 0.075 and RMSE was 0.092. This was –385, 69 %, 0.099 and 0.113 for EQ-5D-3L respectively. The mean observed vs. predicted utilities were 0.572 vs. 0.577 and 0.515 vs. 0.523 for EQ-5D-5L and EQ-5D-3L respectively, for OLS; for BB, these were 0.572 vs. 0.575 and 0.515 vs. 0.518 respectively and for LVDMM 0.532 vs 0.515 and 0.569 vs 0.572 respectively. Less over-prediction at poorer health states was observed with EQ-5D-5L.

**Conclusions:**

The BB mapping algorithm is confirmed to offer a better fit for both EQ-5D-3L and EQ-5D-5L. The results confirm previous and more recent results on the use of BB type modelling approaches for mapping. It is recommended that in studies where EQ-5D utilities have not been collected, an EQ-5D-5L mapping algorithm is used.

## Background

Health Related Quality of Life (HRQoL) is an important outcome from both clinical and economic perspectives. For cancer patients, it can be considered as a measure of the trade-off between survival benefit, toxicity from treatments and the physical and emotional well-being of the patients [1]. HRQoL is also considered to be an important predictor of survival [2]. Furthermore, HRQoL is critical for understanding the economic value of (cancer) treatments, because some cancer treatments are not only expensive but also the clinical benefits are modest and the burden of adverse events is quite high. Therefore, the risk-benefit relationship of cancer treatments can be guided by HRQoL outcomes [3].

One feature of health economic evaluation is the use of generic HRQoL measures to determine patient level health utilities for adjusting clinical outcomes to generate Quality Adjusted Life Years (QALYs) [4]. In some cases, utilities from commonly used generic HRQoL measures such as EQ-5D-3L or EQ-5D-5L are not always available. Therefore, reliance is made on alternative approaches to estimate patient level utilities using ‘mapping’ or ‘cross-walking’ – where a statistical algorithm developed from a condition-specific measure (e.g. such as the cancer specific EORTC-QLQ-C30) is used.

The advantages and limitations of mapping have been discussed in detail elsewhere (Khan, 2014; Brazier, 2010) [5, 6]. Recently Crott (2014), Arnold (2015) and Doble (2015) [7–9] examined the performance of the most common mapping algorithms applied to the QLQ-C30. Several limitations of some of the simpler mapping algorithms from the EQ-5D-3L were noted. These related to untenable assumptions of linearity, homoscedasticity, multimodality, skewness, censoring and an over reliance on R^2^ as the metric of model performance; and in some cases poor over prediction, particularly at poorer health states [5, 7, 8, 10, 11]. Mapping algorithms based on EQ-5D-3L have been shown to consistently over-predict utilities, particularly at poorer health states [5, 6]. In order to address some of the limitations, alternative functional and statistical forms of mapping algorithms were examined (Kharroubi 2007, Crott, 2010, Khan, 2014, Hernandez, 2012, Sabourin et al., 2015) [5, 10–13]. These functional forms in some cases generated improved predictive capability (e.g. Hernandez, 2012, Khan, 2014). In some cases however, changing the functional form did not offer improved prediction over and above simpler models [5, 6]. Moreover, when applied to external data, some of the algorithms performed poorly [7, 8].

In addition to the statistical framework of mapping algorithms, questions have been raised about the usefulness and indeed validity of mapping (Round, 2012) [14]. It is suggested that it is unclear as to what exactly is being predicted from mapping models, because the target is unknown (Round, 2012) [14]. However, this is precisely what a mapping model is supposed to do - to estimate the unknown utilities, which we assume to be ‘knowable’ based on reasonable assumptions. Although this, among other criticisms of mapping are important [5, 6, 15], they are perhaps not strong enough to dismiss mapping altogether. Consequently, about 25 % of health technology appraisal (HTA) submissions to NICE have used mapping (Longworth, 2013) [16] in the UK; while in Australia, this was reported to be about 24 % (Suchffham, 2008) [17]. Moreover, the published mapping models (for the QLQ-C30), suggest the unknown utilities are likely to be ‘knowable’ to some extent because some mapping algorithms have shown to yield close approximates of the target mean utility. Therefore, mapping can serve a useful purpose for estimating patient level utilities and continues to be used in HTAs of cancer drugs for estimating utilities (or sensitivity analyses) despite these criticisms.

Separately, concerns have also been raised about the sensitivity of the EQ-5D-3L and by extension to the derived mapped utilities [18–21]. Most mapping using the EORTC-QLQ-C30 (QLQ-C30) are based on EQ-5D-3L. Given the reported limitations and criticisms levelled against the EQ-5D-3L and the consequent development of the EQ-5D-5L, a mapping algorithm for the EQ-5D-5L appears to be the next logical step in this area of research.

There are two commonly used generic HRQoL measures for determining utilities used in health economic evaluation - EQ-5D-3L and the more recent EQ-5D-5L. The main difference between these two instruments is that the latter has responses measured on a 5 point scale, with many more health states [22]. EQ-5D-3L was suggested as having limited discriminative ability and less power to detect between group differences compared with EQ-5D-5L [22–24]. Research is ongoing as to the best value sets for use with EQ-5D-5L. Meanwhile, an interim scoring is currently available for EQ-5D-5L using a crosswalk algorithm from EQ-5D-3L to EQ-5D-5L.

In this research we compare the performance of three mapping algorithms (from QLQ-C30): a Random Effects linear model, a Beta-Binomial (BB) and a Limited Dependent Variable Mixture Model (LDVMM), for each of two utility measures: EQ-5D-5L and EQ-5D-3L, separately. To our knowledge, no study of mapping compares algorithms from *both* instruments in the same set of patients; and none are available between EQ-5D-5L and QLQ-C30, particularly from a non-small cell lung cancer (NSCLC) patient population. Khan & Morris (2014), using data from a randomized controlled trial (RCT) [5], showed that a three-part BB model performed best amongst other commonly used algorithms. This analysis examines mapping models using data from NSCLC patient in a real world NHS setting. This will offer researchers a way of computing patient-level utilities from the EQ-5D-5L (and EQ-5D-3L) with greater generalizability than a RCT.

## Methods

### Study design

A single cohort prospective (non-interventional) follow up study in 100 NSCLC patients was designed. Patients with histologically confirmed NSCLC gave informed consent (for data collection and follow up) and were followed up during their routine anti-cancer treatment and cancer management for a period of at least 12 months. Patients were recruited between March 2014 and July 2015 from the Liverpool and Clatterbridge Cancer Centre. The trial recieved local ethics approval (Liverpool Central) and research was conducted in compliance with the Helsinki declaration.

EQ-5D-5L, EQ-5D-3L and QLQ-C30 assessments were carried out monthly from registration. EQ-5D-3L and EQ-5D-5L were assessed at least 1 week apart to avoid potential for ‘carry over’. Patients were given the HRQoL forms to take home and they returned them by post or when they attended their next hospital visit. They were instructed to complete the EQ-5D-3L in the first week and the EQ-5D-5L in the second (or third) week of each month.

### Instruments

EQ-5D-3L is widely used for economic evaluation, has 243 health states and for each state, a corresponding utility value is available [5, 6]. In this paper, we use the UK tariffs based on the Time Trade-Off (TTO) method [23]. The raw scores from the EQ-5Ds were converted into an index ranging from -0.549 to 1, where 1 denotes 'perfect' quality of life, 0 for death and values below 0 as states 'worse than death'. EQ-5D-5L consists of five questions identical to EQ-5D-3L (mobility, self-care, usual activities, pain/discomfort, and anxiety/depression), but with an expanded 5 point scale (compared to the 3 point scale of EQ-5D-3L) [25]. These are ‘no problem’, “’slight problems’, ‘moderate problems’ and ‘severe problems’ in all five dimensions, and ‘unable’ in mobility, self-care and usual activities or ‘extreme problems’ in pain/discomfort and anxiety/ depression. The scoring of EQ-5D-5L uses an interim cross-walk based algorithm (UK value sets) between EQ-5D-3L and EQ-5D-5L (Van Hout, 2012) in the absence of a full value set [22, 26].

The EORTC QLQ-C30 is an established instrument for measuring HRQoL in various cancers [27]. QLQ-C30 has 15 domains, scored on a 0 to 100 scale. The scoring consists of 5 function scales: Physical Function (PF), Role Function (RF), Emotional Function (EF), Cognitive Function (CF) and social functioning (SF). There are also 9 symptom scales: Fatigue (FA), Nausea & Vomiting (NV), Pain (PA), Dyspnoea (DY), Insomnia (IN), Appetite Loss (AL), Constipation (CO), Diarrhoea (DI) and Financial Problems (FI); there is also a global health status score (QL). For the global health and function domains, high scores indicate better QoL. For the symptom domains, low scores indicate better symptoms.

### Statistical methods

Three models were used to compare the mapping.

### Linear random effects model

The linear model with a random effect is an extension of the ordinary least squares (OLS) model. One importance difference is that subject level effects are included (sometimes called a mixed effects model). In the context of mapping, because utility scores are observed for each subject on more than one occasion, the responses are not independent. The subject level differences (between subject variability) can be modelled with a random effect. For this reason the model is termed a mixed effects model because variability of utilities occurs between and within subjects. This model is relatively easy to use when applied to an external data set to predict patient level utilities. This is important because, in practice, a mapping algorithm should also have a feature that it can be used practically and as simply as possible. Overly complicated models require more assumptions and hence introduce greater uncertainty. The principle of parsimony should be adopted when developing a mapping model. The model form in a general linear mixed model framework is:

$$ \mathrm{Y} = \mathrm{X}\upbeta + \mathrm{Z}*\mathrm{u} + \upvarepsilon $$

Where β is a matrix with the fixed effects parameters (e.g. the 15 coefficients of the QLQ-C30) and u is a matrix (or vector) with the random (subject) terms and ε is the experimental error term (corresponding to the fixed effects).

### Limited dependent variable mixture model (LDVMM)

A second model proposed by Hernandez et al. [10] belonging to the class of limited dependent variable (LDV) models is the so-called Adjusted Limited Variable Dependent Mixture Model (ALVDMM) [10]. This particular model has several noteworthy features. The first is that it assumes additivity of effects (as in a linear model). The second is that it involves a latent variable that is censored. The censoring occurs (similarly applied in a TOBIT model) because there are considered to be unobservable values. Hernandez et al. [10] noted that since there is a gap in utilities between the values 0.833 and 1 for the EQ-5D-3L, the preferences for health states are in effect ‘cut-off’ on the higher side of values at (or above) 0.833 to a value of 1 (we essentially capture the ceiling effect). That is, if a patient’s (true) utility is >0.833, the instrument (EQ-5D) cannot capture this and we assume a value of 1.

**Fig. 1:**
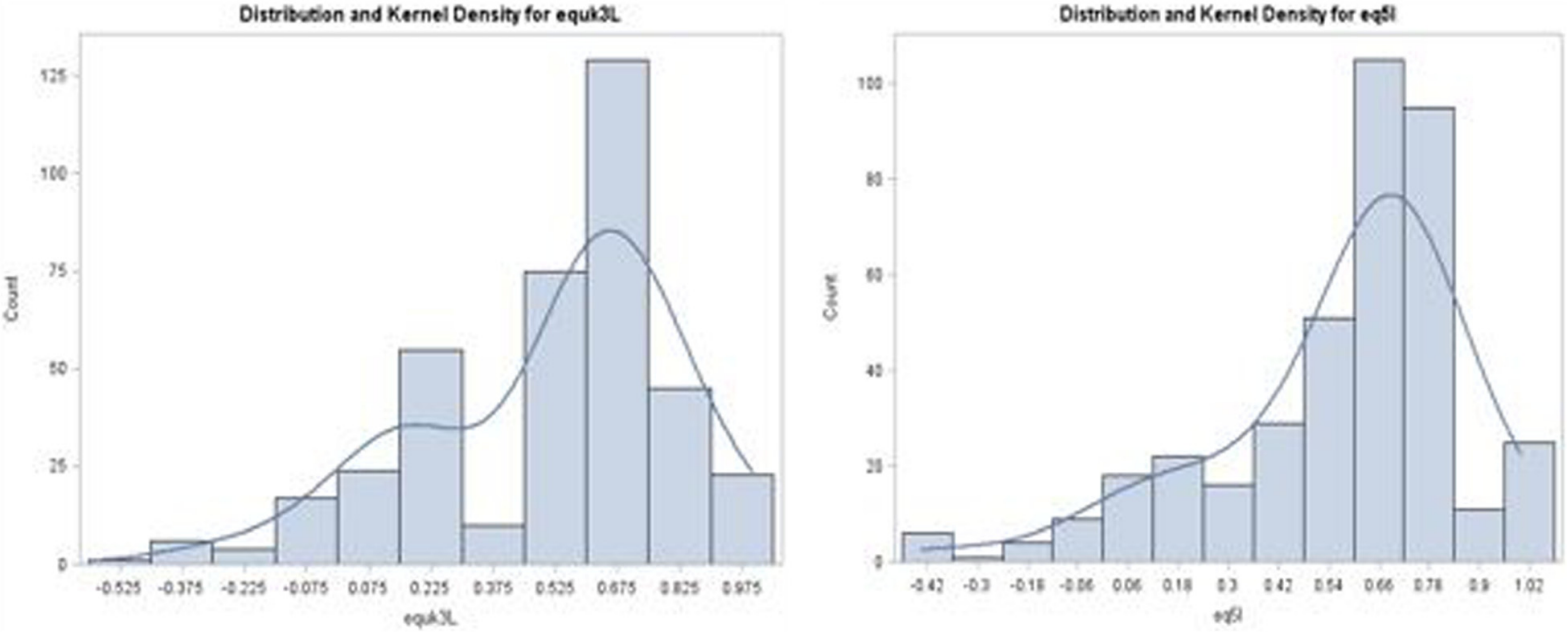
Distribution of EQ-5D-3L (left) and EQ-5D-5L utilities

The LDV type models generate predicted estimates in a more complex way which involve finding the probability that the unobserved (latent) value is above or below the censored threshold value (e.g. 0.833) using the ratio of the probability density function (PDF) to cumulative density functions (CDF). This feature of the LDVs allow the possibility to model the presence of several distributions simultaneously. Hernandez et al. [10] modelled data against the (simpler) health assessment questionnaire (HAQ) in an arthritis population. The greater the number of latent classes, the greater the complexity of interpretation. Application of 3 classes in the context of 15 QLQ-C30 domain parameters is likely to lead to a much more complex latent class structure and therefore two classes (two mixed distributions) are used for both the EQ-5D-3L and EQ-5D-5L in this analysis. This is justified by observing the kernel density estimates which suggest a bimodal distribution for EQ-5D-3L (values between about –0.549 to 0.3 and 0.3 to 1) in this data set (see Fig. 1). For the EQ-5D-5L, the mixture of distributions is not obvious, although there is marked skewness.

The model form for the mixture model used in this context is now described in further detail:

Assuming responses *Y* (i.e. EQ-5D utilities), whose distribution depends on an unobservable random variable *S*; *S* can occupy one of *k* states (*k* = 2 in this example), the number of which might be unknown but is at least known to be finite. Since *S* is not observable, it is referred to as a latent variable. Let π_j_ denote the probability that *S* takes on state *j*. For example, in the case of the EQ-5D-3L for the ALVDMM, j = 1 might refer to values of EQ-5D-3L < 0.833 and *j = 2* would refer to states such that EQ-5D-3L utilities are > 0.833.

Conditional on *S*, the distribution of the response *Y* is assumed to be f_j_(y;α_j_, β _j_| *S = j)*. What this expression (i.e. (f_j_(y;α_j_, β _j_| S = j)) means is that depending on the number of states (S), a model (with a form f_j_(y;α_j_, β) can be used to determine the relationship between Y (the EQ-5D) and a set of predictors, β (e.g. the 15 QLQ-C30 coefficients). For example, for j = 1 (values of EQ-5D-3L between -0.549 and 0.3), the EQ-5D-3L are assumed to follow a Normal distribution. For values between 0.3 and 1 (j = 2), the data can be considered to follow a Beta Binomial (BB) distribution. In another scenario, for j = 1, a Weibull function could be used, and for j = 2 a Normal distribution used; there would 6 parameters to estimate (2 parameters for the Weibull, 2 parameters for the Normal and consequently two mixing probabilities (π_1_ and π_2_), the probability of observations belonging to one or another class. The 6 parameters to be predicted do not include any of the QLQ-C30 predictors (parameters), where a further, 16 parameters are estimated.

The following mixture models were simultaneously fitted:

(i) EQ-5D as a function of 15 QLQ-C30 domain scores (Normal Distribution assumed between -0.549 and 0.30 for example)
(ii) EQ-5D as a function of 15 QLQ-C30 domain scores (Beta Binomial distribution assumed between 0.30 and 1 for example)
(iii) The Mixing probabilities as a function of the 15 QLQ-C30 domain scores (two mixing probabilities which classify observations as belonging to distributions in (i) or (ii))

Clearly, the above modelling approach is complex, perhaps unnecessary and can lead to model non convergence. Its practical implementation as an external algorithm is therefore an important consideration. A transformation may be carried out if specific distributions are assumed (e.g. modelling negative values). For example, for values between -0.549 and 0.30, a Gamma (or Beta Binomial) distribution would not be possible.

Therefore, in this analysis two distributions are considered for modelling:

(i) Assume Normality between -0.549 and <0.30 for the 15 predictor variables
(ii) Assume Beta Binomial between >=30 and 1.0 for the 15 predictor variables

The predicted estimates are determined in a complicated way from the ratio of the CDF to the PDF of the EQ-5D responses and using the estimated mixing probabilities. The mixing probabilities can be interpreted as the ratio of observations belonging to one of two distributions. If the mixing probabilities were 0.5, then 50 % of the EQ-5D-3L might be considered to follow a Normal distribution and the remaining 50 % a different distribution. A useful exposition of finite mixture models can be found in Schlattman (2009) [28].

A maximum likelihood estimation for continuous and discrete response distributions is used based on a dual quasi-Newton optimization algorithm using the SAS® software [29]. A global maxima was sought using initial starting values to search for a local maxima, followed by re-running the model using estimates generated from previous model runs.

### Beta binomial model

For the ALVDMM previously used, censoring occurs for values at 0.833 for the EQ-5D-3L. This is not the case for the EQ-5D-5L, where values between 0.833 and 1 do exist. For this reason (Fig. 1) the distribution of the EQ-5D-5L can be considered appropriate for modelling on a continuous type scale between -0.549 and 1.0 (after a transformation of Y-a/b-a), and therefore the BB model is the third model that is considered for mapping. The details of the BB model are elaborated and discussed in Khan & Morris (2014) [5] and show an improved fit compared with simpler linear and LDV type models (e.g. TOBIT and CLAD).

### Model performance criteria

Several model performance statistics were used including the root mean square error (RMSE) which is a measure of model fit (lower values indicate better fit), mean prediction error, R^2^, mean absolute error (MAE), and percent predicted >1 and < -0.594 were. Chai (2014) argues that the RMSE is more appropriate than the MAE, particularly if the error distribution is Normally distributed [30]. In addition, the Aikakes Information Criteria (AIC) values and percent predicted within a target range (e.g. ±5 %, ±10 %) of the observed values were determined.

### Simulation and cross validation

Multivariate simulation (1,000 simulations using Fleishman methods) [31, 32] were used to test the uncertainty of the models. The method of Fleishman uses higher order moments (e.g. kurtosis and skewness) to generate correlated simulated data regardless of the distribution of each of the original variables. The steps involved in simulation require computing the mean, SD, skewness and kurtosis for each of the observed 15 QLQ-C30 domain scores. Using the Fleishman (1978) [31] power transform:

$$ \mathrm{Y}=\upalpha +\upbeta *\mathrm{Z} + \updelta *{\mathrm{Z}}^2+\upgamma *{\mathrm{Z}}^3, $$

The values of α, β, δ and γ are estimated from randomly generated data Z, normally distributed with mean of zero and a variance of 1 and the observed measures for kurtosis and skewness. The values of α, β, δ and γ are estimated through a process of iteration so that Y can be determined. The derived Y (e.g. 15 QLQ-C30 scores) are simulated (correlated) responses which are not necessarily normally distributed. Khan et al. [5] have shown that the QLQ-C30 scores are unlikely to follow a Normal distribution in most cases.

For each simulated data set, cross validation was used. Half (50 %) of the simulated dataset (randomly selected) was used to develop the mapping model and the other half used to test the model (out of sample predictions). For each realization (i.e. dataset simulated), the model performance statistics (e.g. RMSE and R^2^) were generated and reported. Although, there is no theoretical reason for 50 % of the data used for developing the model, other cut-offs (e.g. 75 % vs 25 %) were also considered.

## Results

**Fig. 2:**
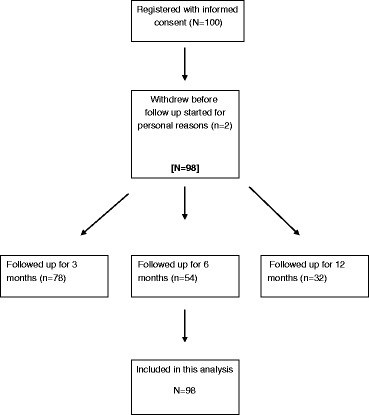
CONSORT

Between March 2014 and July 2015, a total of 100 patients were registered for follow up, out of whom, two patients withdrew before follow up started. Consequently, 98 (98 %) were followed up and included in the statistical analysis; 23 patients (23 %) died during the follow up and 2 patients (2 %) dropped out due to personal reasons (Fig. 2 CONSORT). There were a total of 985 observations (responses) across 98 patients for EQ-5D-5L and EQ-5D-3L HRQoL forms, respectively; HRQoL forms were completed by 97/98 (99 %) patients at baseline; completion rates at 3 and 6 months were 78/98 (79 %) and 41/98 (55 %) respectively. Completion rates were, therefore, similar for all three (EQ-5D-5L, EQ-5D-3L and QLQ-C30) instruments. There were 146 observed health states (5 % of all possible health states) observed with EQ-5D-5L and 62 (26 %) for EQ-5D-3L. The most frequent health states with the EQ-5D-5L were 11111 (6 %), followed by 21222 (5 %), 43533 (3 %) and 31331 (3 %). For EQ-5D-3L these were 21222 (11 %), followed by 22222 (10 %), 22221 (7 %), 22322 (6 %) and 11111 (6 %).

### Demographics

Median age was 69 years (range 39 to 86); 55/98 (56 %) were male, 67/98 (68 %) were ex-smokers and 19/98 (19 %) current smokers. There were 61/98 (64 %) patients who were Easter Co-operative Oncology Group (ECOG) (0-2) and the remaining with ECOG >2; ECOG is used as a measure of well-being (and prognosis), with higher values suggesting poorer prognosis; 15/98 (15 %) were Stage I-II and 83/98 (85 %) were Stage III and higher; Histology subtypes were 43/98 (44 %) with adenocarcinoma and 36/98 (37 %) with squamous cell. The remainder were of varying subtypes (Table 1).

**Table 1:** Baseline and demographics characteristics

	(*N* = 98)
Age (Median years, Range)	69 (39-86)
Gender:	55 (56 %)
Male	43 (44 %)
Female	
Smoking Status	
Current Smoker	19 (19 %)
Ex-Smoker	67 (68 %)
Never	5 (5 %)
Unknown	7 (7 %)
Stage	
I -II	26 (27 %)
III	31 (32 %)
IV	37 (38 %)
Unknown	4 (4 %)
Histology	
Adenocarcinoma	43 (44 %)
Squamous	36 (37 %)
Mesothelioma	5 (5 %)
Other	14 (14 %)

### Performance of EQ-5D-5L and EQ-5D-3L Mapping Algorithms

#### Overall

**Fig. 3:**
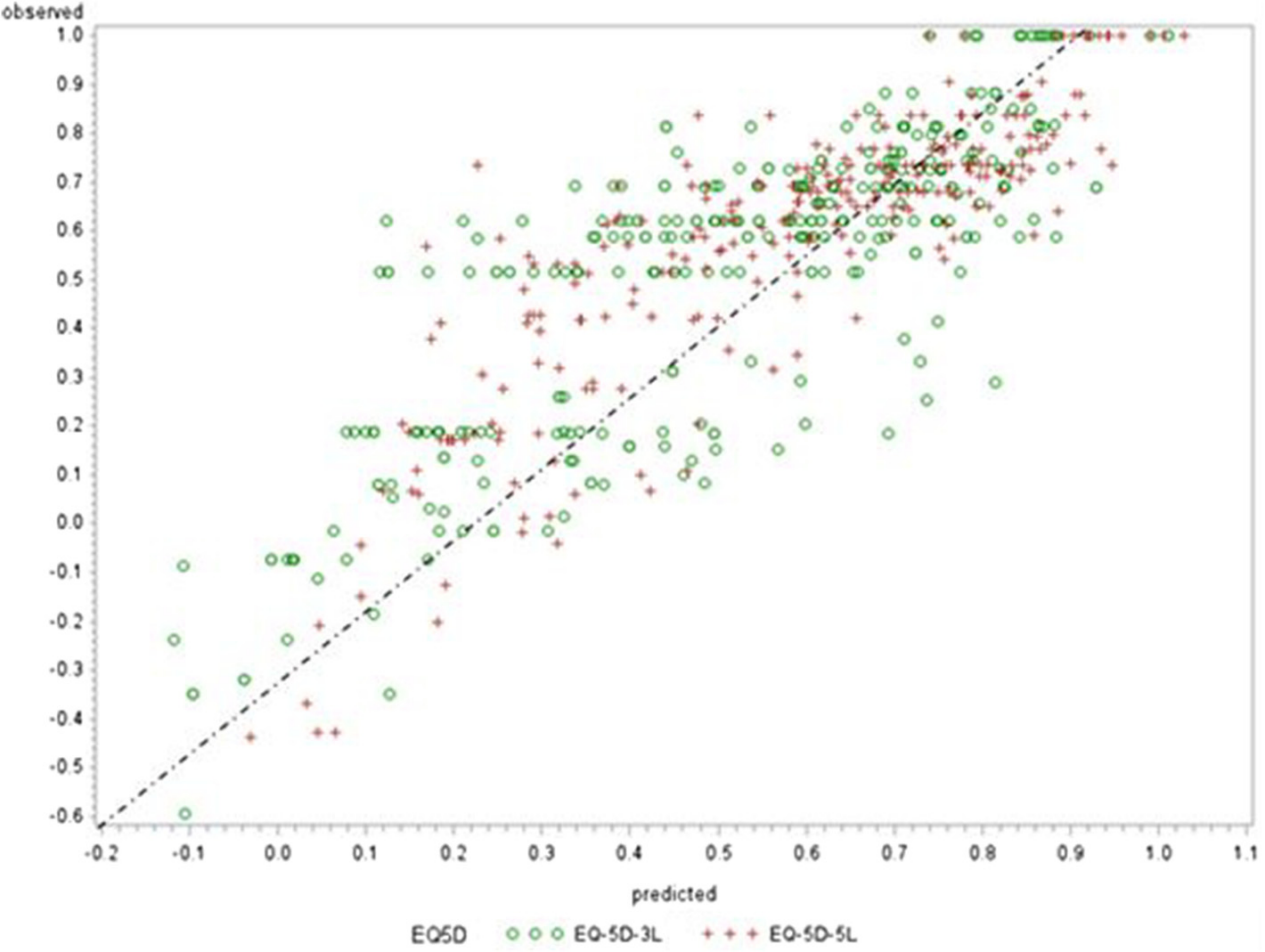
Scatter Plot of Observed vs. Predicted Values (EQ-5D-5L, EQ-5D-3L) – BB Model. Note: at lower utility scores (poorer health), over –predictions is greater with the EQ-5D-3L compared with EQ-5D-5L

The best performing model regardless of EQ-5D-3L or EQ-5D-5L was the BB model (Table 2 & Fig. 3): this had AIC, R^2^, RMSE, MAE and % predicted to within ±5 % and ±10 % of -485.3, 75 %, 0.092, 0.075, 29 % and 59 %; for EQ-5D-3L and were -385.4, 69 %, 0.113, 0.099, 21 % and 47 % for EQ-5D-5L respectively. The BB therefore had good model fit characteristics and predicted more utilities to within ±10 % of the observed value compared to other models, particularly for the EQ-5D-5L.

**Table 2:** Comparison of Model Performance

	EQ-5D-5L			EQ-5D-3L		
	Random effect	Beta binomial	LVDM^a^	Random effect	Beta binomial	LVDM^a^
R^2^	72 %	75 %	70 %	67 %	69 %	67 %
AIC	-365.3	-485.3	-383.2	-291.4	-385.4	-189.1
RMSE	0.152	0.092	0.153	0.183	0.113	0.179
MAE	0.114	0.075	0.115	0.141	0.099	0.139
Predicted Mean (SD)	0.577 (0.241)	0.575 (0.211)	0.569 (0.217)	0.523 (0.252)	0.518 (0.183)	0.532 (0.252)
Observed Mean (SD)	0.572 (0.224)	0.572 (0.224)	0.572 (0.224)	0.515 (0.308)	0.515 (0.308)	0.515 (0.308)
%predicted outside range	<1 %	0	0	<1 %	0	0
Predicted within ±5 %	19 %	29 %	20 %	19 %	21 %	20 %
Predicted within ±5 %	38 %	59 %	42 %	37 %	47 %	35 %

^a^Normal + Beta Mixture

### Random effects model

The performance of the random effects model was comparable to the LDVMM. Table 3 shows the parameter estimates for the 15 QLQ-C30 coefficients. If all scores for the functional domain, Global score and Finance score are assumed to be perfect (i.e. score of 100) and no signs and symptoms are present (i.e. score of 0), the predicted EQ-5D-3L and EQ-5D-5L scores are estimated to be about 0.89 and 0.96 respectively. On the other hand, if symptom and functional scores are the worst possible (scores of 0 and 100 for function and symptoms respectively), the predicted EQ-5D-3L and EQ-5D-5L falls to about 0.10 and 0.09 respectively. EQ-5D-5L therefore predicts better at both extremes Table 5.

**Table 3:** Comparison of model performance of other mixture models

Mixture	AIC	
	EQ-5D-5L	EQ-5D-3L
Normal /Beta	-383.2	-189.1
Normal/Gamma^a^	-250.5	-250.5
Normal/Weibull^a^	-252.4	-128.4
Normal/Log Normal	-242.0	-124.4

^a^model convergence problems resulted in some parameters not estimated and/or mixing probabilities not calculable

### Beta binomial model

Following on from above, the BB can be used to predict the EQ-5D using a standard logit link: P/1-*P* = exp (-α + βX), such that *P* = 1/1 + exp (-α + βX), where P are the predicted EQ-5D and X are the QLQ-C30 scores.

The first step is to predict the EQ-5D using the estimates in Table 4. Setting the functional scores of the EQ-5D-3L to perfect HRQoL for the two function and symptom scores (score = 100 and 0 respectively), the predicted EQ-5D-5L is estimated as:

**Table 4:** Results from statistical modelling (Random effects Model)

	EQ-5D-5L			EQ-5D-3L		
	Estimate	SE	*P*-value	Estimate	SE	*P*-value
Intercept	0.2255	0.09157	0.0142	0.08046	0.08507	0.3450
Physical Functioning	0.006418*	0.000676	<.0001	0.006137*	0.000620	<.0001
Role Functioning	-0.00032	0.000591	0.5935	0.001392*	0.000509	0.0066
Emotional Functioning	0.001871*	0.000554	0.0008	0.001949*	0.000481	<.0001
Cognitive Functioning	-0.00057	0.000491	0.2436	-0.00073	0.000448	0.1024
Social Functioning	0.000387	0.000530	0.4664	0.000516	0.000462	0.2652
Global health status / QoL	-0.00109*	0.000409	0.0082	-0.00043	0.000401	0.2853
Fatigue	0.000324	0.000696	0.6420	0.000993	0.000647	0.1261
Nausea / Vomiting	-0.00041	0.000600	0.4990	0.000276	0.000524	0.5993
Pain	-0.00311*	0.000495	<.0001	-0.00215*	0.000427	<.0001
Dyspnoea	0.000368	0.000464	0.4287	-0.00011	0.000421	0.7915
Insomnia	-0.00017	0.000338	0.6218	-0.00004	0.000313	0.9053
Appetite loss	-0.00030	0.000328	0.3673	0.000341	0.000295	0.2488
Constipation	-0.00013	0.000359	0.7139	0.000524	0.000306	0.0877
Diarhoea	0.001155*	0.000438	0.0087	0.000499	0.000425	0.2409
Financial Problems	0.000345	0.000334	0.3019	-0.00004	0.000297	0.9039

*Statistically significant at the two-sided 5 % level

1/[1 + exp(‐ α + **βX**) = exp[0.2255 + (100 * PF + 100 * SF + … … + 0 * FA …. + 0 * FI)] = 0.983. Hence, the predicted EQ-5D-5L are 0.983, approximating the value 1.00. Table 5 below shows results from scenarios between the 3 models.

**Table 5:** Predicted utilities from 3 scenarios

	Possible QLQ-C30 Score		Predicted	
Model	Function	Symptom	EQ-5D-3L	EQ-5D-5L
Random effects	Best (100)	Best (0)	0.89	0.96
	Worst (0)	Worst (100)	0.10	0.019
Beta Binomial	Best (100)	Best (0)	0.901	0.983
	Worst (0)	Worst (100)	0.097	0.0094
LDVMM	Best (100)	Best (0)	0.884	0.972
	Worst (0)	Worst (100)	0.055	0.008

### LDVM

The LDVM model estimates are more complicated to generate as they involve two distributions and two mixing probabilities. Consequently more than 32 parameters are involved in determining predictions for the best and worst case scenarios (Table 6). The LDVMM also predicts well at extremes, despite similar R^2^ and RMSE to the random effects model (Table 5 and Table 7). However, the LDVMM is much more complex to use as an algorithm. Users would also need to know details of the mixing probabilities as well as make stronger assumptions about the mixed distribution. Other mixtures were also considered but the Normal/Beta mixture offered the best (smallest AIC) fitting model.

**Table 6:** Results from Statistical Modelling (BB Model)

	EQ-5D-5L			EQ-5D-3L		
	Estimate	SE	*P*-value	Estimate	SE	*P*-value
Intercept	-1.51144	0.000060	<0.001	-0.0123	.003893	0.00248
Physical Functioning	0.02254*	0.004666	<0.001	0.08711	.002940	<0.001*
Role Functioning	0.009619*	0.004550	0.03867	0.00421	.002685	0.12215
Emotional Functioning	0.01904*	0.003192	<0.0001	0.00661	.002007	0.00166*
Cognitive Functioning	-0.00633	0.003312	0.06076	-0.00425	.002111	0.04858*
Social Functioning	-0.00013	0.002758	0.97120	-0.00035	.001973	0.85980
Global Health Status / QoL	0.001652	0.002772	0.55344	-0.00197	.001913	0.30724
Fatigue	0.003561	0.005282	0.50279	0.00443	.002979	0.14223
Nausea / Vomiting	0.000452	0.004514	0.92057	-0.00146	.002700	0.59069
Pain	-0.03479*	0.003512	<0.001	-0.01039	.001910	<0.001*
Dyspnoea	-0.00806*	0.002800	0.00553	0.00015	.001759	0.93233
Insomnia	0.002047	0.002388	0.39474	0.00193	.001491	0.20048
Appetite loss	0.005383*	0.002446	0.03161	0.0002	.001415	0.88807
Constipation	0.000454	0.002052	0.82565	0.0014	.001386	0.31650
Diarrhoea	0.000353	0.00274	0.20705	0.00393	.001841	0.03688*
Financial Problems	-0.004324	0.002182	0.07174	-0.00113	.001292	0.38527

*Statistically significant at the two-sided 5 % level

**Table 7:** Results from statistical modelling (LDVMM: Normal + Beta)

	EQ-5D-5L				EQ-5D-3L			
	Normal		Beta		Normal		Beta	
	Estimate	SE	Estimate	SE	Estimate	SE	Estimate	SE
Intercept	0.07353	0.05925	-0.7052	0.4046	0.1032	0.1008	0.1579	0.8373
Physical Functioning	0.008668*	0.000515	0.01394*	0.002851	0.007667*	0.000771	-0.01009	0.005942
Role Functioning	0.000340	0.000439	0.01271*	0.002239	0.000961	0.000943	0.01046*	0.003785
Emotional Functioning	0.002680*	0.000457	0.003145	0.002045	0.001808*	0.000593	-0.00191	0.004717
Cognitive Functioning	-0.00141*	0.000367	-0.00521*	0.001998	-0.00127*	0.000603	0.000919	0.003645
Social Functioning	-0.00085	0.000475	0.001153	0.001935	0.000355	0.000651	0.009044*	0.003982
Global Health Status / QoL	0.000250	0.000236	-0.00051	0.002030	-0.00151*	0.000424	0.001184	0.004713
Fatigue	0.000698	0.000519	-0.00074	0.002929	0.002149*	0.000875	-0.01064	0.006315
Nausea / Vomiting	-0.00063*	0.000368	0.001293	0.002378	0.000278	0.000649	-0.00853	0.006210
Pain	-0.00662*	0.000343	-0.00094	0.001835	-0.00584*	0.000568	0.01325*	0.005386
Dyspnoea	0.001407*	0.000476	-0.00576*	0.001807	0.000640	0.000488	-0.00791*	0.004004
Insomnia	0.000180	0.000239	-0.00156	0.001351	0.000290	0.000384	-0.00656*	0.002875
Appetite loss	-0.00085*	0.000253	0.008535*	0.001406	-0.00081*	0.000388	0.009893*	0.002344
Constipation	0.002190*	0.000261	-0.00215	0.001445	0.001571*	0.000382	-0.00631*	0.003363
Diarrhoea	0.001377*	0.000289	-0.00265	0.001942	0.000749	0.000563	0.005638	0.004770
Financial Problems	-0.00102*	0.000260	0.001778	0.001291	0.000539	0.000337	0.004688	0.002820

*statistically significant at the 2 sided 5 % level

### Health states

EQ-5D-3L prediction by health state were generally as observed in literature (Khan & Morris 2014) [5]: over-prediction at poorer health states. There does however appear to be some evidence that mapping algorithms based on EQ-5D-5L may yield improved predicted utilities at poorer health states. In particular, the BB model showed improved predictions regardless of the instrument.

**Fig. 4:**
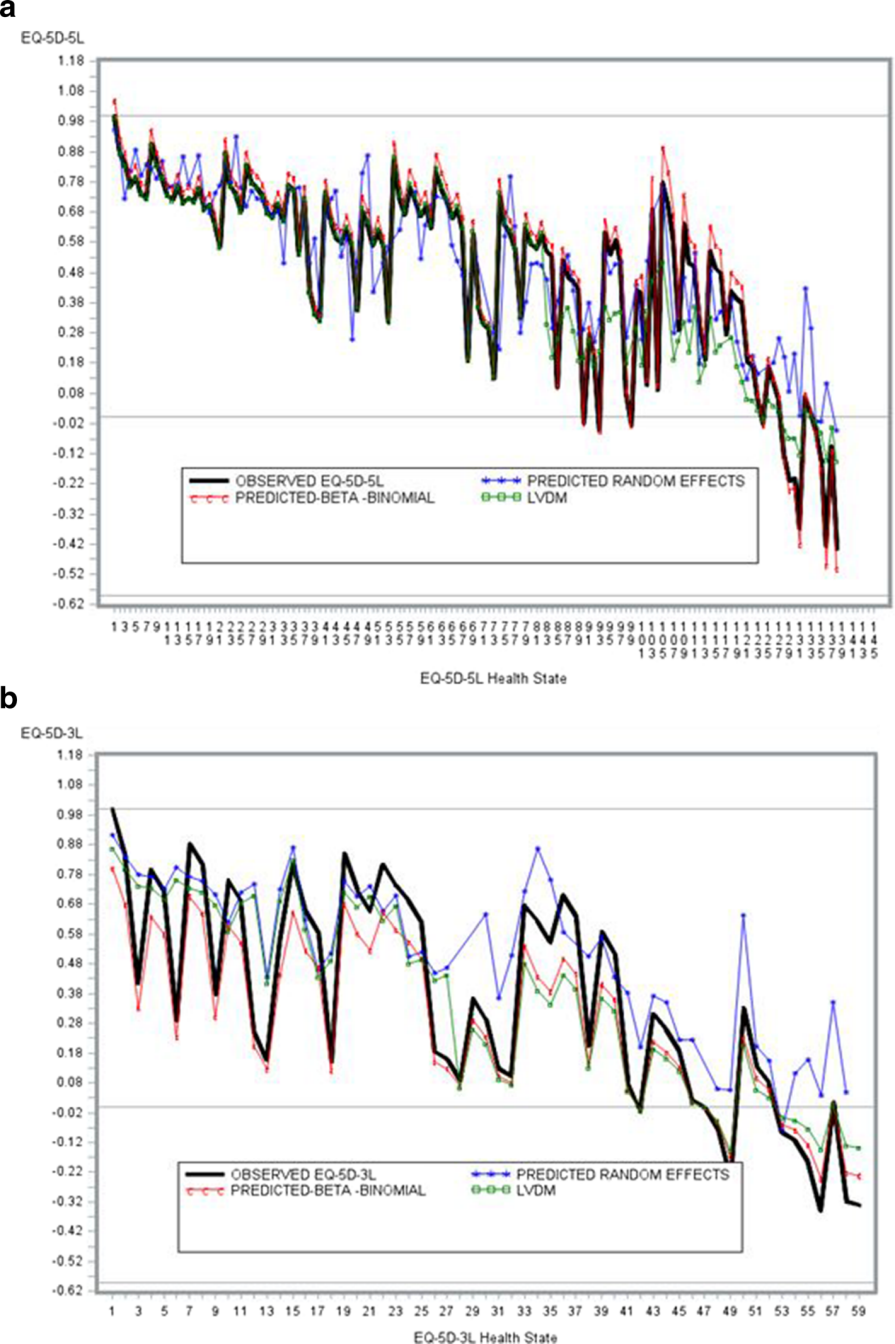
Observed vs. Predicted Values by Health States. **a**. EQ-5D-5L. **b**. EQ-5D-3L

The predictions at poorer health states (Fig. 4) present some interesting findings. Modelling with the LDVMM consisted of a BB and Normal distribution. Values >0.30 were modelled assuming a BB distribution. Predictions at poorer health states (assumed to be -0549 to 0.30) appear slightly worse. Better predictions with the LDVM after EQ-5D values >=0.30 are observed. This supports a BB algorithm as a plausible model for developing a mapping algorithm.

The predicted values are notably worse for the EQ-5D-3L. About 50 % of predicted utilities were over-predictions (higher than the observed value by any amount) with the EQ-5D-5L; for EQ-5D-3L this was 67 %; 93 % vs 97 % of utilities were over-predictions for the EQ-5D-5L vs EQ-5D-3L respectively.

### Simulation and cross validation

Each simulated data set of 985 observations for EQ-5D-5L and EQ-5D-3L were subject to a cross validation using a 50 % random sample (about 492 observations each for EQ-5D-5L and EQ-5D-3L respectively) for the BB model. Hence, a total of 1,000 R^2^, RMSE and mean predicted values were observed (Table 8 and Figures. 5.4 – 5.7). For EQ-5D-5L and EQ-5D-3L respectively, the average (mean) R^2^ from the BB model was 76 % (range 51 % to 89 %) and 68 % (range 38 % to 79 %); RMSEs averaged around 0.099 (range 0.069 to 0.155) and 0.113 (range 0.058 to 0.177). Simulations from the Random Effects and LDVM models showed similar performance but were both worse compared to the BB.

**Table 8:** Results of Simulation and Cross Validation (BB Model)

Algorithm	Parameter	Mean	Lower 95 %	Upper 95 %	Range
EQ-5D-5L	R^2^	0.76	0.69	0.82	(0.51, 0.89)
	RMSE	0.099	0.075	0.121	(0.069,0.155)
	Observed	0.572	-0.018	1.00	(-0.436,1.00)
	Predicted	0.575	0.198	0.950	(0, 1)
EQ-5D-3L	R^2^	0.68	0.58	0.78	(0.38, 0.79)
	RMSE	0.113	0.103	0.120	(0.058, 0.177)
	Observed	0.515	-0.07	1.00	(-0.594, 1.00)
	Predicted	0.518	0.112	0.89	(0, 1)

**Fig. 5:**
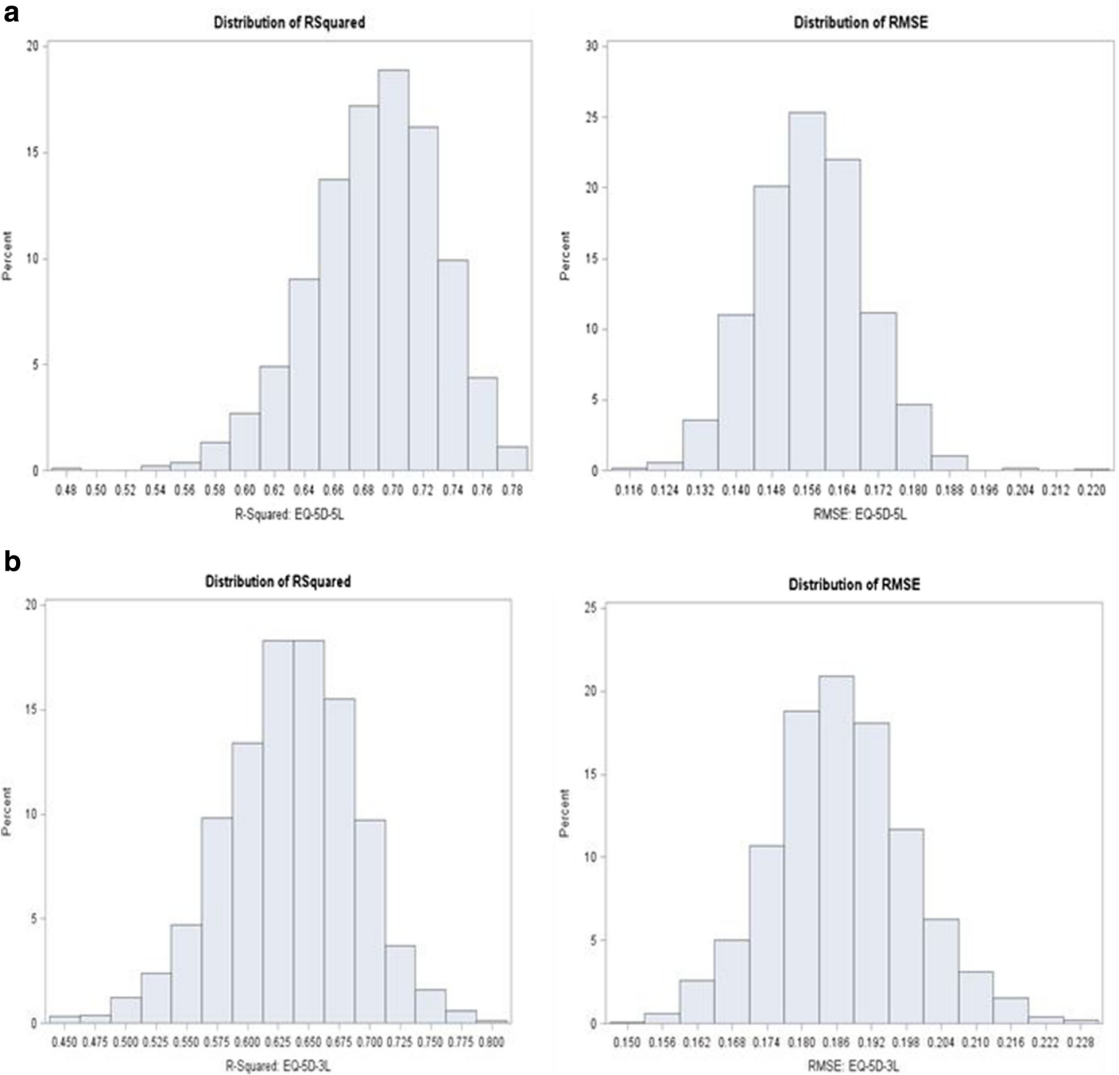
Distribution of R^2^ and RMSE for Each of (**a**) EQ-5D-5L and (**b**) EQ-5D-3L after Cross Validation Models (50 % Holdout Sample) : Random Effects Model

**Fig. 6:**
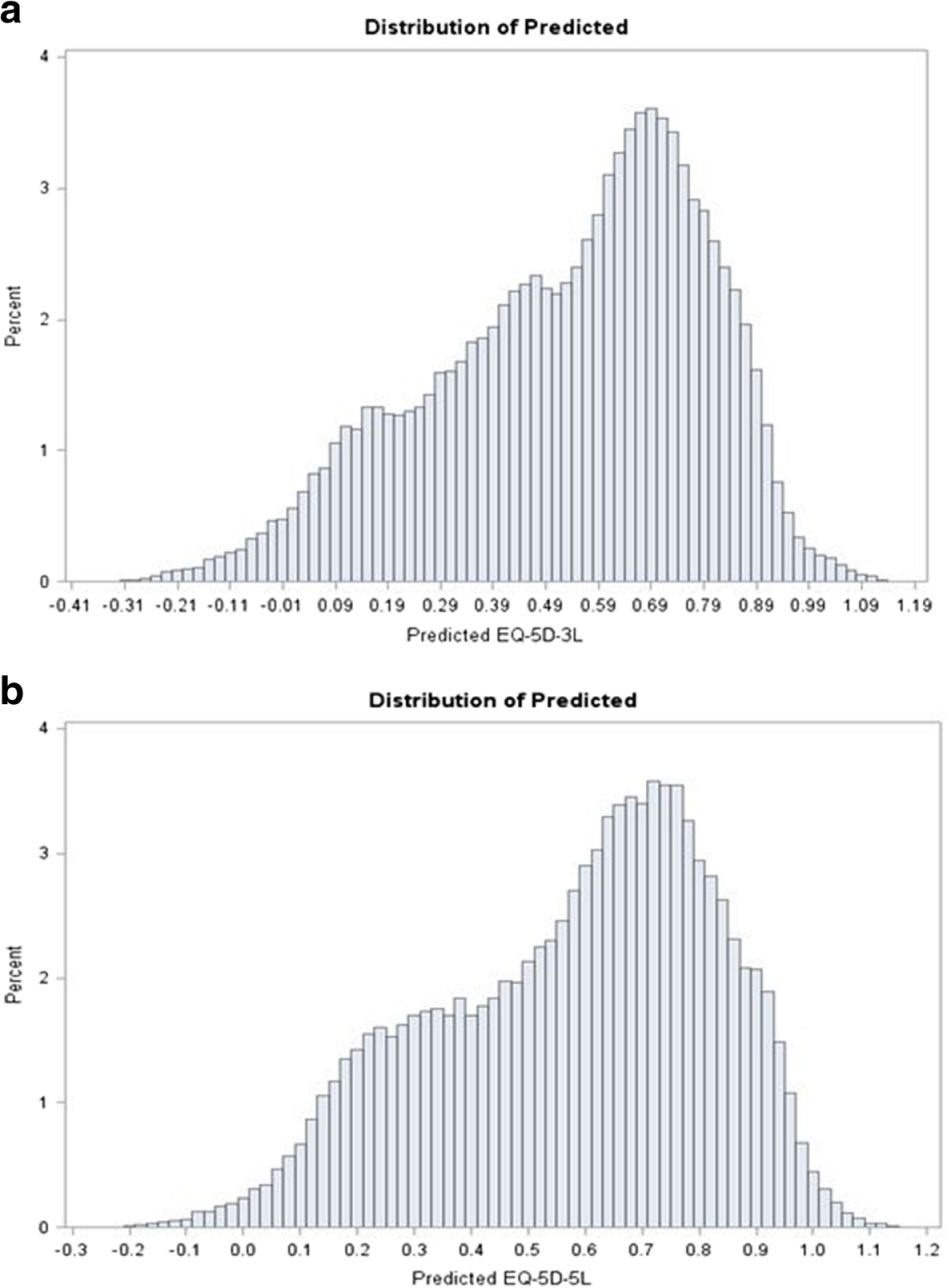
Distribution of Predicted Means (**a**) EQ-5D-5L and (**b**) EQ-5D-3L after Cross Validation Models (50 % Holdout Sample): Random Effects Model. **a**. EQ-5D-3L Predicted Mean. **b**. EQ-5D-5L

**Fig. 7:**
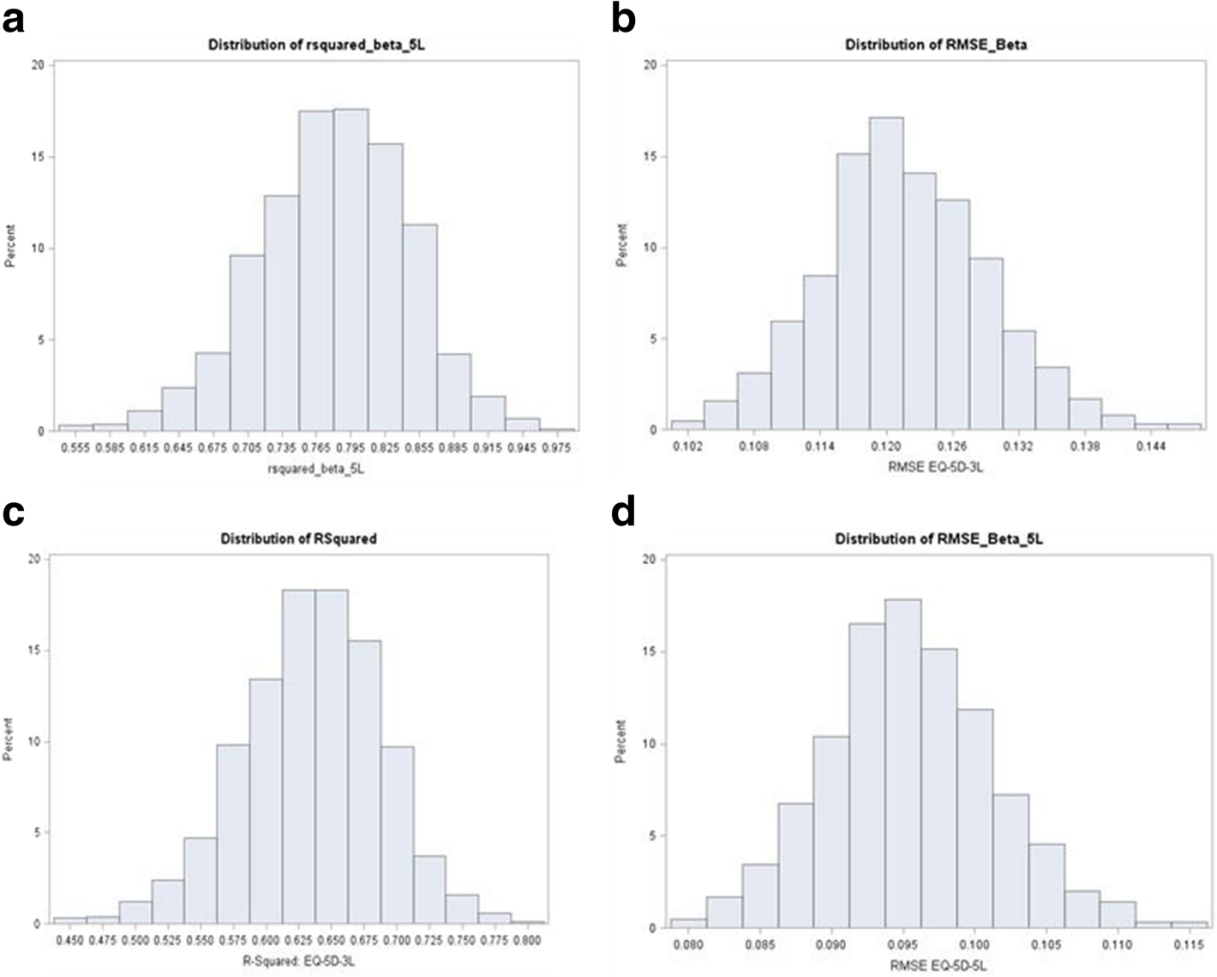
Distribution of R^2^ and RMSE for Each of (**a**) EQ-5D-5L and (**b**) EQ-5D-3L after Cross Validation Models (50 % Holdout Sample): BB Model. **a**. R^2^ EQ-5D-5L. **b**. RMSE 5 L. **c**. R^2^ 3 L. **d**. RMSE 3 L

**Fig 8:**
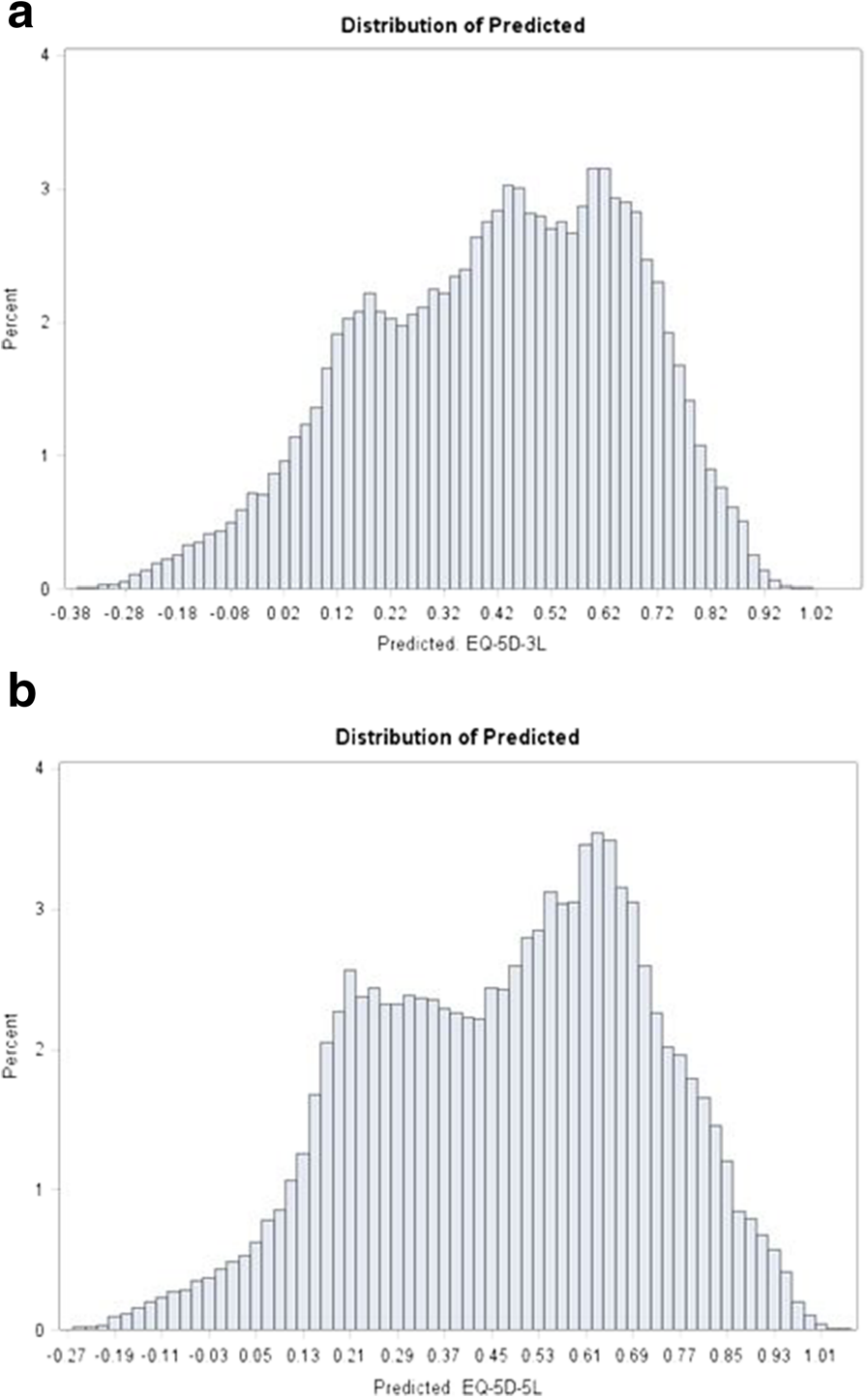
Distribution of Predicted for each of (**a**) EQ-5D-5L and (**b**) EQ-5D-3L after Cross Validation Models (50 % Holdout Sample): LDVMM. **a**. EQ-5D-3L. **b**. EQ-5D-5L

Predicted mean utilities were closer to the observed for the EQ-5D-5L: 0.572 vs. 0.575 whereas, for the EQ-5D-3L these were 0.515 vs. 0.518 (Table 8 and Figs 5, 6, 7 and 8). Hence, out of sample predictions for the EQ-5D-5L appeared more accurate than those of the EQ-5D-3L, particularly with the BB model. When a different cut-off was used (e.g. 75 % to model the data and 25 % for prediction), there were no changes in conclusions.

## Discussion

We have developed and compared three mapping algorithms for the EQ-5D-5L and EQ-5D-3L using contemporary and novel modelling methods. We have shown that EQ-5D-5L may offer better prediction at poorer health states where several previous algorithms with EQ-5D-3L have, by and large, over-predicted. Modest improvements of an algorithm based on EQ-5D-5L over one based on EQ-5D-3L in terms of statistical metrics (e.g. R^2^, percent predicted) have been confirmed with a BB model in this and previous analyses [5]. Young et al. [33] suggested that two-part models may offer a way to predict the different parts of the distribution in the context of mapping with improved performance for handling over-prediction. More recently, Crott [34] confirms the suitability of the BB type models over other models. In this analysis we have confirmed the bimodal nature of the EQ-5D-5L value sets noted earlier (Oppe et al.) [24] (Fig. 6).

This is the first time to our knowledge a mapping algorithm has been developed simultaneously from EQ-5D-5L and EQ-5D-3L in the same lung cancer patients using EORTC-QLQ-C30 and compared with each other in a real world NHS setting. Previous works with the EQ-5D-5L highlighted some of the limitations of the EQ-5D-3L relating to aspects such as bi-modality of utilities and a lack of sensitivity to detect differences between treatment groups [35–37]. Some earlier mapping models did not take this into account. Cheung et al. [25] for example, report an algorithm using the FACT-B in a breast cancer population with R^2^ of around 48 % (AIC was not reported).

In this analysis, over-prediction at poorer health states still exists with EQ-5D-5L, although it is not as marked as EQ-5D-3L. It is yet to be seen whether the final value sets (Oppe et al.) [24] currently being developed and validated will impact predictions at poorer health states. The reasons for over-prediction may be due to several factors, including the functional form of the model, the range of the scale (5 point vs 3 point scale), number of health states and other clinical characteristics. Khan & Morris [5] previously suggested over-estimates at poorer health states may be related to other factors such as poorer prognosis. Preliminary evidence of this is shown by observing the relationship between ECOG performance and EQ-5D utilities (Table 9). It is possible a further complexity is required in the modelling by using the joint distribution of utilities and other outcomes (e.g. Adverse events) to model the QLQ-C30 scores.

**Table 9:** Utilities and ECOG relationship

ECOG	Mean EQ-5D-5L		Mean EQ-5D-3L	
	Observed	Predicted	Observed	Predicted
0	0.706	0.736	0.675	0.702
1	0.625	0.638	0.589	0.600
2	0.502	0.493	0.489	0.437
3	0.317	0.331	0.273	0.284
4	-0.024	0.237	0.067	0.199

In this study, the EQ-5D-5L and 3L assessments were taken close together in time. Therefore, there may be some concern about ‘carryover’ or recall bias. To check this, we determined whether health state responses were recorded similarly. For example, if a response of 11112 was observed for EQ-5D-3L, we checked whether this was also observed for EQ-5D-5L (responses >3 are not possible for EQ-5D-3L). We noted that for 15 of the 146 (EQ-5D-5L) health states, the responses for EQ-5D-5L and EQ-5D-3L were the same - for example, patients with responses of 11111 to both EQ-5D-5L and EQ-5D-3L in 18 of the 985 (pairs) of observations (<2 %). In the vast majority of cases the responses were different. This suggests that patients did not recall the previous responses and the presence of carryovermay be unlikely.

There are several limitations of this research. The first is that this is a small sample size with relatively few health states, although the sample size is larger than the algorithm reported by Kontodimopoulous (2009) [38]. Secondly, inferences need to be restricted to a similar NSCLC population until further evidence emerges of wider applicability across tumour types. Thirdly, external validity was not possible in an independent data set and therefore cross-validation was used as a ‘second best’ accompanied by simulation for out of sample predictions. Fifthly, insufficient numbers of events were available for reliable computation of QALYs and therefore the impact on QALYs could not be reliably observed at this time (a sufficient number of events are not yet available for this to be estimated reliably). Finally, the values of the EQ-5D-5L are cross-walked from the EQ-5D-3L and are therefore subject to uncertainty. However, in the absence of a readily identified set of value sets, and given that the EQ-5D-5L is being used in current clinical research, using the EQ-5D-3L cross-walk sets should be considered acceptable in the interim.

Despite these limitations, this is the first mapping algorithm for the EQ-5D-5L using real world data with enhanced generalizability outside the RCT context. That further research is required, is consequently inevitable.

## Conclusion

Mapping algorithms developed from EQ-5D-5L appear to provide improved estimates of utilities compared with EQ-5D-3L, particularly at poorer health states. Two part models fit the data well and this result confirms earlier and more recent work. It is recommended that in studies where EQ-5D utilities have not been collected, an EQ-5D-5L mapping algorithm is used.

### Panel: research in context

#### Systematic review

We carried out an extensive review of the literature before designing this study. At the time no comparison of HRQoL responses across several important HRQoL instruments were made in a lung cancer patient population, particularly the EQ-5D-3L and EQ-5D-5L. Understanding HRQoL continues to be an important aspect of managing NSCLC patients and this research will be valuable for future economic evaluations and understanding the way different HRQoL instruments measure utility.

### Interpretation

We have demonstrated that the EQ-5D-5L can be mapped from the EORTC-QLQ-C30 successfully. Our findings suggest that the EQ-5D-5L may be a preferred choice of mapping in NSCLC patients due to its higher R^2^, improved prediction in general and at poorer health states, where EQ-5D-3L algorithms have shown to over predict. The results of this study may lead to wider use of the EQ-5D-5L.
